# Wanted: special-purpose robots automating life science wet lab workflows

**DOI:** 10.4155/fso.15.64

**Published:** 2015-11-01

**Authors:** Ming Li

**Affiliations:** 1 Biogen, Inc, 250 Binney Street, Cambridge, MA 02142, USA

**Keywords:** laboratory automation, life science, special-purpose robots, wet lab

Compared with manufacturing and service industries, the life science R&D industry in general is lagging behind in terms of utilizing large-scale industrial automation for productivity, capacity and quality improvements. Granted, the exploratory and dynamic nature of life science R&D dictates that for most life science R&D projects that come and go, there is not a fixed procedure to begin with, and there is no way to predict how a project is going to end up either. This uncertainty makes automation of general life science R&D workflows difficult. Still, life scientists around the world are working hard to isolate, extract and automate the portions of the workflow that can be automated [1]. Over the past decades, much progress has been made in the automation of analytical separation and detection, data acquisitions and downstream data extraction and processing. Relatively speaking, the progress of automating life science wet lab workflows has been much slower, due largely to the lackluster tools available.

## The downsides of general-purpose liquid handling robots

Since a common denominator of life science R&D wet lab workflow is liquid, current robot manufacturers all aim to maximize the applicability of their products to assorted life science workflows (translation: maximizing profits) and offer general-purpose liquid handling robots (GPLHR). This makes their internal robotic products R&D relatively simple, but while the goal of GPLHRs is to suit all applications, they in fact please no one. For ordinary end-user groups, those robots are just too much of a hassle – difficult to use, crash often and the results vary [2–4]. Since there are no commercially available liquid-handling robots that tailor to their specific workflows, much postacquisition customization and development needs to be done either by internal staff, vendor staff or both, if they wish to automate more than just a few isolated steps of their experiments. Customization, development and maintenance costs aside, the process is slow, there is often no guarantee of the quality of such custom work and quality and success varies from one organization to another. For those end users’ management, it is often a struggle to see through the clouds of the capital expenditure, dedicated automation FTE investment [2], slow customization and slow adoption among ordinary scientists to find out whether the robots are worth the investment [3,4]. For the robot manufacturers, because the barrier of entry for GPLHRs is relatively low, competition is fierce. Due to slow adoption of these robots, the robot manufacturers are not fully realizing the huge life science market potential. Also, they might become the failure of their own success if the repair/maintenance service, technical support and most notoriously and unpredictably, custom development demand cannot keep up with their sales.

In short, there is much to be desired from a contemporary GPLHR. To give a few specific examples:

Tip management. Disposable tips eliminate the sample contamination issue and are more advantageous than syringes. Contemporary GPLHRs have internal tip counting libraries to help keep track of tip usage. While the idea is great, it is not doing a good enough job. The end users do not need to know where the next available tip is located, and they should not be expected to interfere with tip management. Their only responsibility for tips should be to ensure there are enough assorted tips for their experiments. Everything else should be handled automatically by the robot. A much improved tip feeding mechanism would be vertical barcoded stacks of assorted tip types on a carousal under the deck, and a 2D laser mesh tip sensing grid sitting underneath the deck surface at the microtiter plate opening to determine in real time the remaining tip count and the last tip location for the tip rack. On the deck, there should be only one tip rack opening for all tip-related operations. This would save much deck space for experimental purposes.
Upstream and downstream workflow hardware integration. Liquid transfers are important parts of web lab workflows, but they are intimately intertwined with other liquid-related operations such as solution preparation, capping/sealing containers, vortexing, centrifugation, decapping/desealing containers, nitrogen drying, plate reading, among others. Big pharmaceutical companies are integrating these devices with GPLHRs postmarket to make the system fully walk-away. Not every company can afford that kind of internal development. Why do the robot manufacturers not do this at the factory?
Workflow-specific software. Postmarket customization and development in the form of extensive scripting by user/vendor staff to suit particular workflows actually in a sense indicate that GPLHRs are half-finished products. Customization and development work needs to be done at the manufacturer to begin with and scripting is not good enough. Again, users do not need to know and should not be expected to know the inner-workings of the automation equipment. The scripting layer should be eliminated altogether. Workflow-specific user interface and business logic, including straightforward enterprise IT data integration configuration options, should all be done at the factory.
Intelligence. Common causes for GPLHR system halts and irrecoverable crashes include fatal tip/insufficient liquid errors, which can all be eliminated with additional intelligence built into the system. To continue with the tips example above, the tip-sensing 2D laser grid would report the status (number of tips and last tip position) of the partial tip rack in real time on demand. The system would combine that information with the number of same type full tip racks (read from the barcodes) available in the carousal, and keep a record of how many of that type of tips are available. If the system also knows/computes at the beginning of a run how many of this type of tips would be needed for the experiment, then the system could alert the user to add more tips if it foresees shortage. A common sense for every lab scientist is that one cannot get more liquid out of a container than what was put in there prior. And yet, current GPLHRs do not have that simple intelligence. Conceivably, the robot could keep an exhaustive internal database of container ID, well positions, volumes of liquids, implement some sort of aliquot safety mechanism [MARS] and if the entire experimental procedure is known at the beginning of a run, computes liquid transfer scheme and warn users of potential insufficient liquid errors before the experiment even starts.
The above wish list is by no means comprehensive and could go on.

## Integrated specialized robots automating life science workflows

In summary, what life science R&D scientists really want are turn-key intelligent automation solutions that automate most, if not all, of their wet lab workflows without too much tinkering on their part [3,5–6]. Addressing the above list of issues/wishes would be a good start toward that direction.

In recent years, there have been relevant advances in that direction both in terms of hardware and software. Hardware wise, most recently a European automation lab is trying to tie assorted stand-alone analytical chemistry instruments/apparatuses together on a common hardware platform to achieve fully automated analytical chemistry workflows. Earlier this year, a UK/US-based company just introduced to the market their ingenious robot that automates aqueous solution preparation starting from solids weighing. Software wise, there have been several publications on attempts to automate majority portions of relatively well-defined bioanalytical wet lab workflows [7–11]. It is important to note that the concepts are entirely applicable to many other life sciences R&D wet lab workflows [6,12].

What we need are ultimate vertical integrators who would take on the tasks of consolidating assorted hardware and software products and advancements into integrated specialized robots that would automate large vertical sections of life science wet lab workflows. The tasks are not as impossible as they may seem. For each specific wet lab workflow, the lab hardware needed is usually limited. Therefore, such integration could start with a few workflows and then expand into/integrate more workflows. Along the way, a stream of robotic products with increasing complexity and expanding functionalities could be introduced to the market.

## Conclusion

Life science R&D labs need more automation and scientists want automation to work for them, not the other way around. Ideal life science R&D wet lab robots should automate entire workflows, as opposed to mere liquid transfer steps. To achieve that, vertical integration of workflow-specific hardware and development of workflow-specific software are needed. There is no question that it would be challenging product R&D for robot manufacturers, but the effort would definitely be worth it. The fact that life science R&D industry have invested heavily in and have so far put up with half-finished products (i.e., current crop of GPLHRs) shows the dire need for automation. The underlying marketing message to robot companies is clear: if robot companies can come up with a better product, the life science R&D industry would embrace it and pay for it. The market potential for such robots would be tremendous. The race to special-purpose robots that would reshape the life science R&D wet labs is on.
